# Self-Assembly of Polymer-Modified FePt Magnetic Nanoparticles and Block Copolymers

**DOI:** 10.3390/ma16165503

**Published:** 2023-08-08

**Authors:** Frank Hartmann, Martin Bitsch, Bart-Jan Niebuur, Marcus Koch, Tobias Kraus, Christian Dietz, Robert W. Stark, Christopher R. Everett, Peter Müller-Buschbaum, Oliver Janka, Markus Gallei

**Affiliations:** 1 Polymer Chemistry, Faculty of Natural Sciences and Technology, Saarland University, Campus C4 2, 66123 Saarbrücken, Germany; frank.hartmann@uni-saarland.de (F.H.); martinbitsch@live.de (M.B.); 2 INM—Leibniz-Institute for New Materials, Campus D2 2, 66123 Saarbrücken, Germany; bart-jan.niebuur@leibniz-inm.de (B.-J.N.); marcus.koch@leibniz-inm.de (M.K.); tobias.kraus@leibniz-inm.de (T.K.); 3 Colloid and Interface Chemistry, Faculty of Natural Sciences and Technology, Saarland University, Campus D2 2, 66123 Saarbrücken, Germany; 4 Physics of Surfaces, Institute of Materials Science, Technical University of Darmstadt, Peter-Grünberg-Straße 2, 64287 Darmstadt, Germany; dietz@pos.tu-darmstadt.de (C.D.); stark@pos.tu-darmstadt.de (R.W.S.); 5 Chair for Functional Materials, Department of Physics, TUM School of Natural Sciences, Technical University of Munich, James-Franck-Straße 1, 85748 Garching, Germany; christopher.everett@ph.tum.de (C.R.E.); muellerb@ph.tum.de (P.M.-B.); 6 Heinz Maier-Leibnitz Zentrum (MLZ), Technical University of Munich, Lichtenbergstraße 1, 85748 Garching, Germany; 7 Inorganic Solid-State Chemistry, Faculty of Natural Sciences and Technology, Saarland University, Campus C4 1, 66123 Saarbrücken, Germany; oliver.janka@uni-saarland.de; 8 Saarene, Saarland Center for Energy Materials and Sustainability, Campus C4 2, 66123 Saarbrücken, Germany

**Keywords:** block copolymers, self-assembly, nanoparticles, nanocomposites, anionic polymerization

## Abstract

The fabrication of nanocomposites containing magnetic nanoparticles is gaining interest as a model for application in small electronic devices. The self-assembly of block copolymers (BCPs) makes these materials ideal for use as a soft matrix to support the structural ordering of the nanoparticles. In this work, a high-molecular-weight polystyrene-*b*-poly(methyl methacrylate) block copolymer (PS-*b*-PMMA) was synthesized through anionic polymerization. The influence of the addition of different ratios of PMMA-coated FePt nanoparticles (NPs) on the self-assembled morphology was investigated using transmission electron microscopy (TEM) and small-angle X-ray scattering (SAXS). The self-assembly of the NPs inside the PMMA phase at low particle concentrations was analyzed statistically, and the negative effect of higher particle ratios on the lamellar BCP morphology became visible. The placement of the NPs inside the PMMA phase was also compared to theoretical descriptions. The magnetic addressability of the FePt nanoparticles inside the nanocomposite films was finally analyzed using bimodal magnetic force microscopy and proved the magnetic nature of the nanoparticles inside the microphase-separated BCP films.

## 1. Introduction

An emerging class of smart materials is magnetoactive soft matter [1]. The responsiveness of polymer composite films with incorporated magnetic nanoparticles makes them ideally suited for sensor applications, magnetic data storage [2,3], and electromagnetic shielding [4]. The use of block copolymers (BCPs) as scaffolds for the incorporation of magnetic nanoparticles (NPs) can, for instance, combine the structural control at the nanometer-length scale, or the mechanical properties of polymers, with the functionalities of nanoparticles within nanocomposite films [5,6]. BCPs have the advantage of assembling into spherical, cylindrical, bicontinuous, or lamellar structures. The generated morphology of the nanostructure depends on the volume percentage of each block, the architecture of the polymer blocks, and the concentration of NPs [7]. A “bottom-up” approach can control the positional ordering of nanoparticles in a self-assembled BCP morphology [8], but self-assembly is still a non-conventional method to prepare nanostructured hybrid materials. NPs can be seen as homopolymers, which can be blended with the specific BCP phase and swell the domain size [9]. Nonetheless, the addition of NPs induces a high risk of defect structures, which have to be overcome by controlling the processing conditions. The addition of NPs also introduces additional surface area from the particles and eventually adds stabilizing molecules or ions [7]. In general, different methods of film preparation, like spin coating [5,10], dip coating [4], nanopatterning [11], evaporation-induced self-assembly (EISA) [12], printing [13], solution casting [9], and photochemical etching [14], have been applied. 

Small organic molecules can easily assemble into one or more of the polymer phases if the interaction is favorable [15]. For example, Xu et al. showed the possibility of inducing phase separation in a miscible BCP by adding different surfactant molecules through supramolecular interactions [16]. Just like with organic molecules, inorganic particle precursors can also be assembled within a BCP microdomain. Afterward, NPs can be generated in situ from inorganic precursors, via chemical reduction, thermal decomposition, or vapor deposition methods. A major drawback of this method is the lack of control over particle size and inter-particle ordering [8]. In comparison to organic molecules, inorganic nanoparticles normally show weak dispersibility within organic polymer matrices because of the intrinsic incompatibility resulting from their different chemical natures [4]. Therefore, chemical modifications of the nanoparticles to tend towards a higher affinity and mixing capability with the polymer blocks are gaining attention in current research [3]. Attractive interactions between the modified nanoparticles and—at least—one part of the BCP can lead to a high load of nanoparticles in the respective polymer domain. Van der Waals interactions give only a small enthalpic contribution to obtain an up-concentration and self-assembly of nanoparticles in at least one BCP domain. Stronger enthalpic influences could be generated via ionic interactions, dipole–dipole interactions, or hydrogen bonds [8]. Song et al. [17] used ZrO_2_ nanoparticles coated with gallic acid, and the resulting H-bonding interaction with polyethylenoxid (PEO) was used to create nanocomposites with a high reactive index contrast. The authors also used the faster self-assembly of bottlebrush graft copolymers instead of linear block copolymers with fewer polymer chain entanglements in order to obtain the nanocomposite within minutes. With a high-volume percentage of nanoparticles, the authors observed a change in the morphology, from a cylindrical to a lamellar structure [17,18]. Hammond et al. [19] did not change the structure, but rather the orientation of the cylinders by adding rod-shaped magnetic nanoparticles of spindle-type hematite (α-Fe_2_O_3_) to a BCP in a magnetic field. The cylindrical domains of the polymer were oriented parallel to the NP rods, which were aligned perpendicular to the magnetic field.

A second major aspect of the self-assembly of nanoparticles in BCPs is the entropic effect, especially for low enthalpic interactions. Larger particles, which were coated with one type of polymer, were located at the center of the suitable domain, so the polymer chains would rather move apart than stretch around the particles, which maximizes the translational entropy [8,20,21]. Smaller particles are more likely to be separated at the inter-material dividing surface (IMDS) [20]. This effect is determined by the fraction of the diameter of the particle *d* to the length of the domain *L*. A low *d*/*L* (typically *d*/*L* < 0.15 [20,21]–0.20 [22]) leads the particle to the A–B-dividing surface, thereby reducing the A–B interactions Therefore, the polymer becomes less stable towards order–order and order–disorder transitions [20]. Bockstaller et al. [22] used the size selectivity of particle separation in block copolymers to produce nanocomposites with two different types of particles. Large SiO_2_ particles were localized inside a lamellar domain of PEP in a poly(styrene-block-ethylene propylene) copolymer (PS-*b*-PEP), while small Au particles were located at the IMDS. The localization of particles at the interface of both polymer blocks can also be driven by coating the polymers with a mixture of both homopolymers [3,21]. The addition of NPs with the same, or larger, sizes as the polymer matrix could lead to unconventional morphologies, through stabilizing a non-equilibrium structure [23]. The resulting structure of the nanocomposite depends not only on the size and volume fraction of the block copolymer and the particles, but also on the polymer architecture [3]. In comparison to the prior results, calculations by Matsen and Thompson resulted in no general preference for smaller particles to the IMDS, and they claim that the driving forces of the material interface are more complicated [24].

The incorporation of PS-functionalized gold nanoparticles in poly(styrene-block-2-vinylpyridine) PS-*b*-P2VP already showed the expected assembly of the particles within the PS domains. The influence of the particle concentration on the microstructure of the block copolymer was already proven, but the solvent casting process led to a height-dependent concentration and, therefore, an inhomogeneous morphology inside the film [9]. In comparison, Yeh et al. [25] used CdS nanoparticles with mercaptoacetic acid as a surfactant to embed nanoparticles in the hydrophilic poly(4-vinylpyridine) (P4VP) domain of a PS-*b*-P4VP block copolymer through a simple solvent-annealing process. The authors shifted the microstructure of PS-*b*-P4VP, from a cylindrical to a lamellar structure, by adding the nanoparticles and analyzing this effect via transmission electron microscopy (TEM), atomic force microscopy (AFM), and small-angle X-ray scattering (SAXS). The influence of nanoparticles on the self-assembly and the morphology of block copolymers matched the theoretical calculations [26,27].

Magnetic nanoparticles can be incorporated into amphiphilic block copolymers without surface modification to form magnetic micelles and vesicles [28]. Moreover, the incorporation of two different inorganic particles into one micelle, where all different properties can be addressed, is also known in the literature [29]. The incorporation of unmodified particles into block copolymer films has also been reported, but additives are required to enable specific nanoparticle–polymer interactions [30]. Another approach is the reduction of an inorganic metallic precursor in a micellar solution and subsequent EISA approach to obtain ordered nanoparticle structures in a block copolymer matrix [12].

Within this work, the influence of different contents of PMMA-functionalized FePt nanoparticles on the self-assembly of an ultrahigh molecular weight block copolymer, PS-*b*-PMMA, forming lamellae domains was analyzed via TEM and SAXS. The focus was to study different loadings of particles followed by observation of loss of structural order with increasing particle loading. These investigations were performed using TEM and SAXS measurements. As another novelty of this work, we investigate the magnetic addressability of the FePt nanoparticles within the PMMA domain using bimodal magnetic force microscopy.

## 2. Experimental Section

### 2.1. Materials

Solvents and reagents were purchased from ABCR, Sigma-Aldrich (Merck, St. Louis, MI, USA), Fisher Scientific (Hampton, NH, USA), VWR (Radnor, PA, USA, TCI Europe (Eschborn, Germany) or Acros Organics (Geel, Belgium) and used as-received unless otherwise stated. For anionic polymerization, the following purification steps were performed: Dried tetrahydrofuran (THF) was obtained by adding *sec*-butyllithium (*s*-BuLi, 1.3 M in hexane) and 1,1-diphenylethylene (DPE) to THF (HPLC grade, Merck, Darmstadt, Germany) in a round-bottomed glass ampule until a dark red color was obtained (diphenylhexyllithium). The liquid was degassed via freeze–pump–thaw cycles and stored under vacuum until use. LiCl was dispersed in dry THF, treated with *s*-BuLi, and stirred overnight. The solvent was removed under reduced pressure and the LiCl was stored in a glovebox until use. Before use, THF was cryo-transferred to an ampule, which was transferred to a glovebox. LiCl was added, followed by additional treatment with *s*-BuLi. The solution was stirred for at least two hours before use. DPE (97%, Sigma-Aldrich, St. Louis, MI, USA) was treated with *n*-BuLi until it turned to a dark red color, then cryo-transferred and stored in a glovebox until use. Methanol was dried using a molecular sieve (3 Å), degassed via freeze–pump–thaw degassing, then cryo-transferred and stored in a glovebox until use. Styrene and methyl methacrylate (MMA) were pre-dried by stirring with calcium hydride. Before use, styrene and MMA were freshly cryo-transferred twice, the first time to an ampule containing dibutylmagnesium; for MMA, trioctylaluminium was used as a drying reagent. All purifying steps of the reagents used for anionic polymerization were carried out using the usual Schlenk technique on an all-glass high-vacuum line or, when indicated, in a glovebox (MBRAUN, UNIlab^plus^ ECO, Garching, Germany) operating with nitrogen gas (5.0) and equipped with a cryostat. For nanoparticle functionalization, MMA was passed through a basic alumina column to remove the inhibitor before use. Toluene and triethylamine (TEA) were dried over CaH_2_ and freshly distilled before use. *N*,*N*,*N*′,*N*″,*N*″-pentamethyldiethylenetriamine (PMDETA) and anisole were degassed and stored under argon in a glovebox. Pt(acac)_2_ and Fe(CO)_5_ in octadecene were used to prepare the FePt NPs, as previously published by Li et al. [31] and Cao et al. [13]. Further details on the coating can be found in the Supporting Materials.

### 2.2. Methods

Size-exclusion chromatography (SEC) was performed using a 1260 Infinity II (Agilent Technologies, Santa Clara, CA, USA) system. THF (HPLC grade, flow rate 1 mL min^−1^) was used as the mobile phase on an SDV column set from polymer standard service (PSS, Mainz, Germany) (SDV 10^3^ Å, SDV 10^5^ Å, SDV 10^6^ Å, 5 µm) with a PSS SECurity^2^ RI/UV detector. Calibration was performed using polystyrene (PS) standards from PSS. PSS WinGPC UniChrom V 8.31 was used for data acquisition and analysis of the measurements.

Nuclear magnetic resonance (NMR) spectra were acquired using a Bruker Avance II 400 spectrometer (Bruker, Karlsruhe, Germany) with a 9.4 T Ultrashield Plus Magnet and a BBFO probe, and referenced using the solvent signals [32]. MestReNova 14.2.0 (Mestrelab Research, Santiago de Compostela, Spain) was used to process and analyze the spectra.

Fourier-transformed infrared (FTIR) spectra were acquired on a Bruker Alpha II FTIR setup in attenuated total reflection mode (ATR) with spectra output in transmittance. All spectra were processed using OPUS 8.5 (SP1) software (baseline correction) and Origin2020b (normalized).

Small-angle X-ray scattering (SAXS) experiments were performed on a Xeuss 2.0 system (Xenocs SAS, Grenoble, France). The incident beam from a copper *K_α_* source with a wavelength *λ* = 0.154 nm was collimated and focused on the sample with a spot size of 0.25 mm^2^. The 2D scattering intensity patterns were collected using a Pilatus 300 K detector (pixel size 172 × 172 μm^2^) located at a sample-detector distance of ~2500 mm, calibrated using a silver behenate standard. For each measurement, the acquisition time was 3600 s. Since no evidence of anisotropic scattering was observed, the scattering patterns were azimuthally averaged to obtain the scattered intensity *I* in dependence on momentum transfer *q* = 4*π* × sin(*θ*/2)/*λ*, with *θ* being the scattering angle. The polymer films with thicknesses of ~0.25–0.5 mm were placed directly in the beam, i.e., without using a sample container, and measured under vacuum conditions at room temperature. FePt NPs dispersed in toluene were measured in a borosilicate capillary. For this purpose, neat toluene was measured as a reference and subtracted from the data. The corrected scattering curve obtained for the FePt NP dispersion was fitted using a model for spheres with a Gaussian size distribution, given by the function

(1) $$I\left(q\right)=A\int_{0}^{\infty }G\left(R_{NP},r\right)r^{6}F^{2}\left(q,r\right)dr+I_{bkg}$$

with *A* being a scaling factor and *I*_bkg_ a constant accounting for background scattering. *F*(*q*,*r*) is the form factor of monodisperse spheres, given by

(2) $$F\left(q,r\right)=3\frac{\sin\left(qr\right)-qr\cos\left(qr\right)}{{\left(qr\right)}^{3}}.$$

To account for the polydispersity of the sphere size, a Gaussian size distribution is assumed, given by

(3) $$G\left(R_{NP},r\right)=\frac{1}{\sigma \sqrt{2\pi }}\exp\left[-\frac{1}{2\sigma^{2}}{\left(r-R_{NP}\right)}^{2}\right]$$

with *R_NP_* the mean radius of the nanoparticles, and *σ* the standard deviation of the size distribution.

For TEM analysis, ultrathin sections (40 nm) were prepared with an ultramicrotome (Reichert Ultracut by Leica Microsystems, Wetzlar, Germany) and placed on a copper grid. A thin carbon layer was added via vacuum decomposition to obtain stable films without charging during transmission electron microscopic examination. Bright-field TEM images were acquired using a JEOL JEM-2100 LaB_6_ electron microscope (JEOL, Tokyo, Japan) operating at 200 kV acceleration voltage; 0.14 nm lattice resolution equipped with a Gatan Orius SC1000 camera (AMETEK, Berwyn, PA, USA) (binning 2; 1024 × 1024 pixels).

Bimodal magnetic force microscopy in the repulsive interaction regime to localize the magnetic nanoparticles within the polymeric material was applied to the samples. For this purpose, an atomic force microscope (Cypher, Asylum Research, Oxfords Instruments, Santa Barbara, CA, USA) in combination with a magnetic cantilever (type: PPP-MFM) (Nanoandmore GmbH, Wetzlar, Germany) was used. The cantilever with force constants *k*_1_ = 3 N/m and *k*_2_ = 17 N/m (calibrated using the thermal noise method [33]) was magnetized with a strong permanent magnet before measurement. To accomplish the bimodal configuration, the first two eigenmodes (*f*_1_ = 71.1 kHz, *f*_2_ = 449.8 kHz) of the cantilever were simultaneously excited photothermally with the BlueDrive system of the microscope. The amplitude of the first eigenmode was used for the topographical feedback whereas the second eigenmode amplitude and phase served for the structural and magnetic visualization, respectively. The free oscillation amplitudes were set to *A*_01_ = 98 nm and *A*_02_ = 1 nm, which were calibrated by recording an amplitude-versus-distance curve on a sapphire substrate. To assure imaging in the repulsive interaction regime, an amplitude setpoint ratio *A_sp_*/*A*_01_ of 0.6–0.7 was set. Images were taken at a 1 Hz scan rate with 512 × 512 pixels.

Powder X-ray diffraction (PXRD) patterns of the pulverized samples were recorded at room temperature on a D8-A25-Advance diffractometer in Bragg–Brentano *θ*-*θ*-geometry (goniometer radius 280 mm) with a copper *K_α_* source with a wavelength of *λ* = 0.15406 nm). A 12 µm Ni foil, serving as a *K_β_* filter, and a variable divergence slit were mounted at the primary beam side. A LynxEye detector with 192 channels was used at the secondary beam side. Experiments were carried out in a 2*θ* range of 20 to 110° with a step size of 0.013° and a total scan time of 2 h. The recorded data were analyzed using the Bruker TOPAS 5.0 software, and the observed reflections were treated by single-line fits.

## 3. Block Copolymer Synthesis

### Synthesis of PS_42_-b-PMMA_58_^312^

An amount of 0.55 mL (0.50 g, 4.8 mmol, 2400 eq.) of freshly purified styrene was added to an ampule containing dried THF and 10 eq of LiCl, and the polymerization was started by rapid addition of 1.4 μL (0.002 mmol, 1 eq.) *s*-BuLi (1.3 M in hexane) at −78 °C via a Hamilton syringe with thorough mixing of the solution. The color changed from colorless to yellowish and the reaction was completed with stirring for 2 h. Then, 0.7 μL (0.7 mg, 0.004 mmol, and 2 eq.) of DPE was added, and the color changed to red at room temperature. An aliquot of 2 mL was taken, treated with a drop of methanol, and precipitated under the air atmosphere by adding methanol. The reaction solution was cooled to −78 °C in a cryostat and 0.60 mL (0.56 g, 5.58 mmol, 2790 eq.) of freshly purified MMA was quickly added with vigorous stirring. After 16 h, the polymerization was terminated by adding a drop of methanol. The mixture was warmed to room temperature, precipitated in methanol, and dried at 40 °C in vacuo. For purification, the polymer was dissolved in THF and precipitated in 2-propanol and dissolved twice in cyclohexane, and precipitated in *n*-hexane. The final polymer showed a nearly monomodal distribution in SEC analysis.

**SEC PS (vs. PS):** *M_n_ = *144,000 g mol^−1^; *M_w_* = 147,000 g mol^−1^; *Đ* = 1.02.

**SEC PS-*b*-PMMA after purification (vs. PS):** *M_n_ =* 312,000 g mol^−1^; *M_w_* = 329,000 g mol^−1^; *Đ* = 1.05.

**^1^H-NMR** (400 MHz, 300 K, CDCl_3_ and δ in ppm): 7.2–6.2 (ar-H PS, 5H); 3.7–3.5 (O-CH_3_ PMMA, 3H); 2.1–0.7 (backbone).

## 4. Preparation of Polymer Films

A standard protocol for solvent annealing of polymer films for TEM has been extended to place the particles into a BCP matrix. For example, to prepare a film containing 0.1 wt% of the functionalized particles, 99.2 mg of the BCP was first dissolved in 0.75 mL of toluene in a 4 mL vial. Then, 100 µL of a 1 mg/mL particle dispersion was added and the dispersion was mixed using a laboratory shaker. After a few hours, the sample was dried at room temperature for 48 h. The drying process was completed under vacuum for one day at 40 °C. Finally, the hybrid film was temperature-annealed in a 4 mL vial at 130 °C for 3 days under a nitrogen atmosphere. The films were sectioned by ultramicrotomy and analyzed by TEM and SAXS.

## 5. Results and Discussion

**Figure 1:**
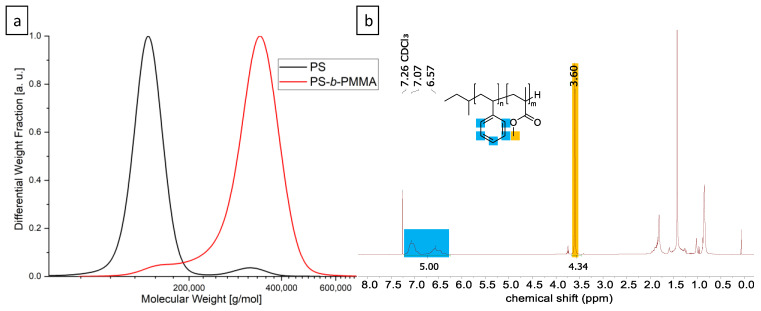
Analytical data of the UHMW PS-*b*-PMMA block copolymer. (**a**) SEC was measured in THF vs. PS standards; (**b**) ^1^H-NMR was measured in CDCl_3_ and referenced to the solvent peak.

To probe the expected assembly of the PMMA-functionalized FePt-NPs in a hybrid block copolymer film, a high molecular weight PS-*b*-PMMA block copolymer was first synthesized. The higher the molecular weight of the polymer, the larger the domain sizes and the easier the nanoparticles will fit into the self-assembled morphology. The polymer was synthesized by sequential anionic polymerization in THF at low temperatures. In detail, styrene was polymerized with *s*-BuLi and later end-functionalized with DPE to prevent an attack on the carbonyl group of MMA for the second block [34]. In the production of high molecular weight block copolymers, problems usually arise with PS homopolymer residues, since small amounts of impurities in the added MMA lead to termination reactions [35]. Fractional precipitation is a common method for the purification of BCPs, taking advantage of the different solubilities of homo- and diblock copolymers and the influence of the larger chain length of the respective block copolymer. Successful purification was verified by SEC (Figure 1a) and the ratios of PS and PMMA were calculated from the corresponding NMR signals obtained by ^1^H NMR spectroscopy in deuterated chloroform (Figure 1b).

**Figure 2:**
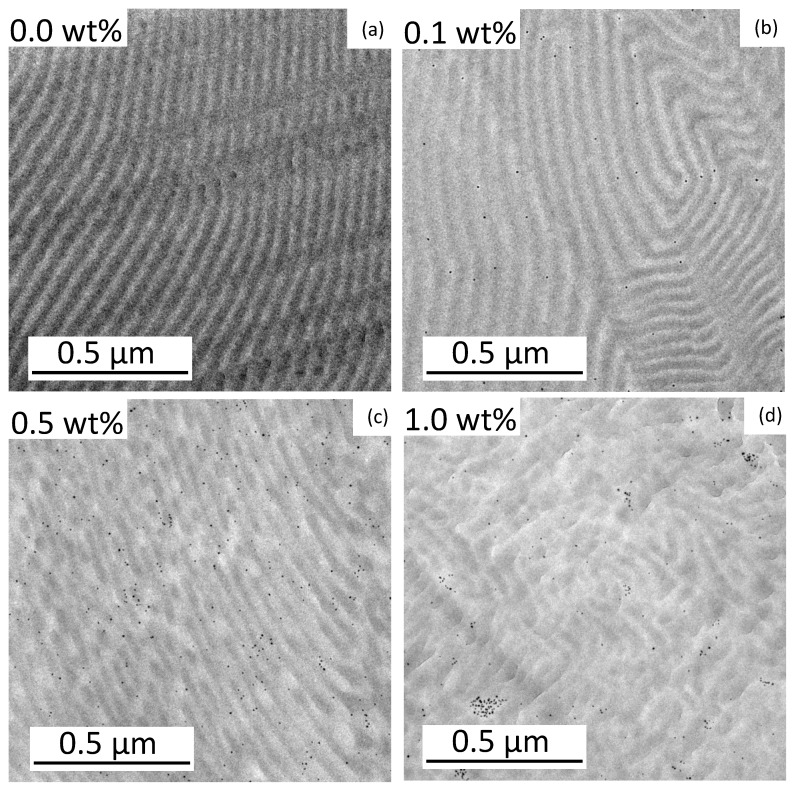
TEM images of the BCP films with FePt-NPs compositions of 0 wt% (**a**), 0.1 wt% (**b**), 0.5 wt% (**c**), and 1.0 wt% (**d**). Within the morphology, the PS domains appear electron opaque (dark) and the PMMA domains are light. The NPs are represented by dark dots.

**Figure 3:**
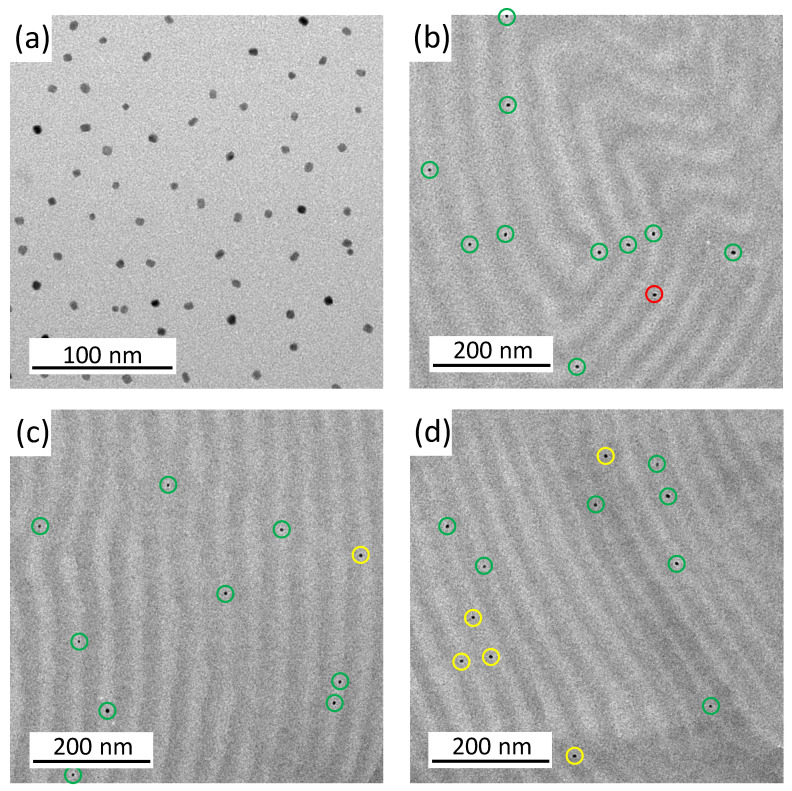
TEM images of the BCP films with 0.1 wt% FePt NP. Figure (**a**) shows the pure FePt NPs, while Figure (**b**–**d**) are different images of the lamellar PS-*b*-PMMA morphology with 0.1 wt% FePt NPs. The particles are marked with a green ring in the light PMMA phase, red in the dark PS phase, and yellow on the boundary.

The pure BCP features a total molecular weight of 312 kg mol^−1^ (*Đ* = 1.05) and a volume fraction of 55.4 vol% PMMA. A film of pure BCP was prepared in toluene for morphology analysis. After fabricating thin slices by ultramicrotomy and carbon desorption using a sputter coater, TEM imaging revealed the expected lamellar morphology (Figure 2a). The aromatic ring system of PS provides the higher electron contrast in TEM and, hence, the PS domains appeared electron opaque (dark). The domain size of the PMMA phase was measured at 20 different points and is 19 nm with a standard deviation of 3 nm. The size of the FePt nanoparticles was also determined by measuring 30 different particles in the TEM with 6.1 ± 0.6 nm (Figure 3a). This results in *d*/*L* = 0.32. These relatively large particles are expected to be in the center of the PMMA phase. Smaller particles (*d*/*L* < 0.2) would be placed at the IMDS [22]. The total thickness of the prepared polymer films is between 0.22 mm and 0.42 mm. The influence of different particle concentrations on the self-assembly of the BCP is shown in Figure 2.

The pure BCP film shows a well-ordered lamellar morphology. The addition of 0.1 wt% FePt NPs already led to a stronger curvature of the lamellae. In the case of particles with 0.5 wt%, the lamellar structure was broken up and clear phases became visible. At 1.0 wt% NPs, the regular structure was almost lost and just a few curved lamellae were visible. The small particle size should reduce the stability of order–order and order–disorder transitions [20]. At higher fractions of the particles, they were more likely to stick together and form larger clusters (Figure S1). The existing attraction between the NPs led to an agglomeration that could not be completely avoided [6]. The clusters also minimized the contact area between the copolymer layer and the particles [20], but at the low NP composition of 0.1 wt%, the particles were well separated and most likely appeared to be located within the PMMA domains. The placement of PMMA-functionalized NPs in the PMMA phase of a block copolymer is expected due to an enthalpic penalty in the PS phase. Enthalpic interactions (and, hence, the Flory–Huggins interaction parameter χ) between the particles and the PMMA phase should be χNP/PMMA = 0 because they are identical. The PS phase repels the PMMA phase and the particles and, therefore, χNP/PS = χPS/PMMA [3]. This behavior was analyzed at a higher magnification as shown in Figure 3. Each particle was labeled with a ring that is green for a particle in PMMA, red in the PS phase, and yellow on the boundary of both phases.

The ordering preference of the 0.1 wt% FePt NPs in the light PMMA phase is directly visible in Figure 3. More TEM images (Figure S1) showed a statistic of only 3 particles (or 4.0%) over 75 particles in the dark PS domains. A total of 43 NPs (or 57.3%) are located in the center of the PMMA domain and 29 particles (or 38.7%) are located at the interface between both phases, but still preferentially located on the PMMA domain side. Thus, 96.0% of the PMMA-functionalized NPs in the PMMA phase. The particle size is well above the boundary between large and small particles, but the particles are slightly more likely to be in the center of the PMMA domain, which is to be expected for larger particles.

Switching to stronger H-bonding interactions between a BCP phase and the functionalization of the NP might be useful to increase the NP concentration. Song et al. [17] implemented up to 30 wt% of ZrO_2_ NPs into a bottle-brush BCP using a solution casting method and H-bonding interaction for photonic nanocomposites. Konefał et al. [4] used a dip-coating process involving H-bonding interactions and even improved the long-range order of the morphology by adding Fe_3_O_4_ NPs. The use of H-bonding interactions will be difficult to sustain when completely non-polar polymers are required for polymer film applications.

**Figure 4:**
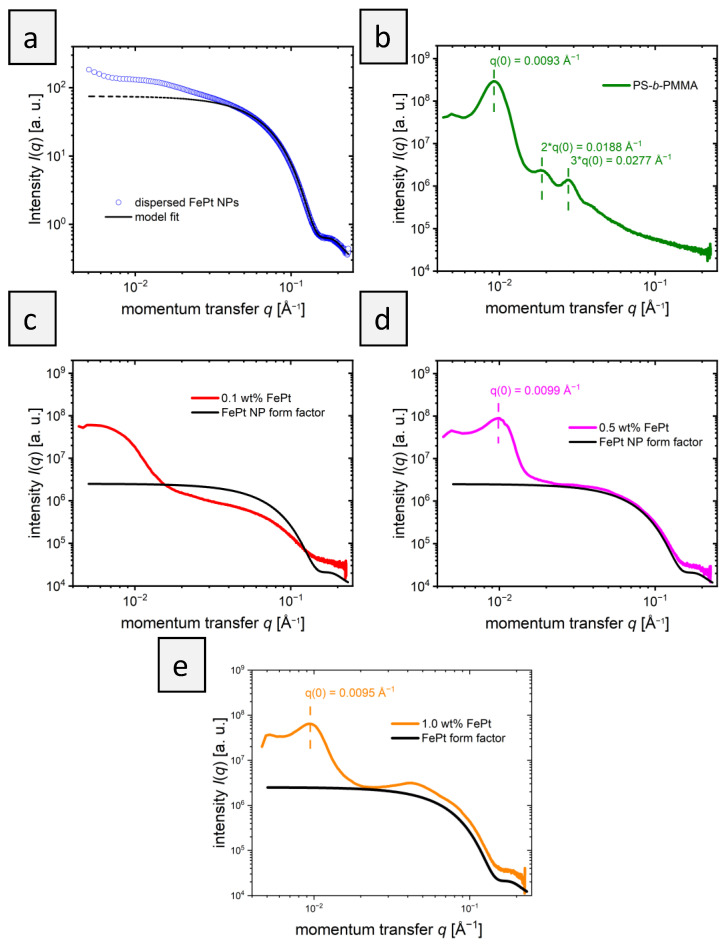
SAXS curves of the PS-*b*-PMMA films with FePt particles. (**a**) FePt dispersed in toluene. The solid line shows a fit according to Equation (1), the dotted line is an extrapolation to low *q*-values. (**b**) PS-*b*-PMMA film in the absence of NPs. (**c**) 0.1 wt% FePt-NPs in PS-*b*-PMMA, (**d**) 0.5 wt% FePt NPs in PS-*b*-PMMA, and (**e**) 1.0 wt% FePt NPs in PS-*b*-PMMA. In (**c**–**e**), the solid black lines denote the form factor of the FePt NPs.

The internal structure of the polymer films was also analyzed by small-angle X-ray scattering (SAXS). First, the scattering of the pure FePt-NPs (Figure 4a) in toluene was analyzed to determine the shape and size of the particles. Fitting the scattering curve with a simple model for polydisperse spheres (Equations (1)–(3) in Section 2.2) neglects the contributions from aggregates at small *q* values. It confirms the spherical shape of the nanoparticles and revealed a mean radius, *R*_NP_, of 2.8 nm and a standard deviation of 0.5 nm. This value agrees with the TEM values, where a diameter of 6.1±0.6 nm was received.

The scattering curve of the pure BCP (Figure 4b) confirms the lamellar morphology with a primary Bragg peak at *q*_0_ = 0.0093 Å^−1^ and secondary Bragg peaks at 2*q_0_ and 3*q_0_ [36]. The repeat distance of the morphology is calculated as 2π/*q*_0_ = 68 nm. This corresponds to the size of a PS and a PMMA lamella, calculated to be 51 ± 4 nm measuring 20 different lamellae using TEM. The larger repeat distance found by SAXS is believed to be due to the larger measured volume compared to the small section used for TEM. This would suggest that the morphology of pure BCP is not the same over longer distances. Figure 4c shows the scattering curve of the BCP film with 0.1 wt% nanoparticles. The nanoparticles embedded in the BCP films dominate the obtained scattering pattern, as shown by the comparison of the scattering pattern with the form factor of FePt-NPs (Figure 4c). Features arising from the morphology formed by the block copolymers are less pronounced than in pure BCP: Only a very broad increase in intensity at ~0.006 Å^−1^ is evident. This finding indicates that the block copolymer morphology is less ordered in the presence of NPs, as could also be concluded from TEM data. The scattering curve of BCP films with 0.5 wt% NPs is shown in Figure 4d. While the scattering of the particles is still dominant, a primary Bragg peak is visible at 0.0099 Å^−1^. For block copolymer films with 1 wt% NPs (Figure 4e), the primary peak is also visible but shifted slightly to 0.0095 Å^−1^. These peak positions correspond to a repeat distance of 63 nm for 0.5 wt% FePt and 66 nm for 1.0 wt% FePt, which is slightly smaller than that of the pure BCP morphology with 68 nm but in the same range. The exact position of the peaks might be slightly affected by the form factor of the particles. The size of the PMMA and PS domains cannot be correctly determined by TEM in Figure 2b–d when particles are added due to the large standard deviation in measuring domain sizes. This trend is caused by the aforementioned decrease in stability towards order–order and order–disorder transitions with added particles and increasing curvature [20].

While the SAXS curve of PS-*b*-PMMA with 0.1 wt% unexpectedly showed no primary structure factor peak, indicating that a repeat distance cannot be determined, higher particle concentrations showed a slight shrinkage of the repeat distance compared to the pure polymer film. The observation of a decreasing overall domain size in SAXS does not support the hypothesis that the addition of NPs to the PMMA phase leads to its expansion. The addition of PMMA homopolymer in a blend system would expand the PMMA phase, which was also expected for the NPs [9]. The SAXS results show that the phases change only slightly in size upon the addition of NPs. Presumably, however, the addition of NPs leads to the formation of a random, instead of a lamellar structure, in the absence of NPs, as also shown by TEM (Figure 2c,d). While TEM indicates a weak remaining order in small areas, SAXS shows a general decrease in structural order over a large sample volume that was probed. The presence of weak secondary structure factor peaks cannot be excluded, since they would overlap with the form factor of the FePt NPs.

In addition, a comparison of the SAXS patterns of PS-*b*-PMMA with embedded NPs with the form factor of NPs gives insights into the distribution of NPs in the films. While at 0.1 and 0.5 wt% the scattering patterns of PS-*b*-PMMA with embedded NPs and the NP form factor follow similar trends above 0.3 Å^−1^, an additional structure factor at ~0.4 Å^−1^ at 1 wt% is discernible. This implies that at 0.1 and 0.5 wt%, the NPs are well-distributed in the film, whereas at 1.0 wt%, clusters of NPs formed. This is in good agreement with the results from TEM, shown in Figure 2.

**Figure 5:**
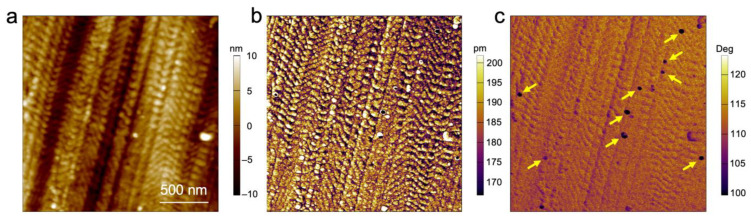
Bimodal magnetic force microscopy of the block copolymer structure with embedded FePt nanoparticles (highlighted with yellow arrows). (**a**) Topography image, (**b**) amplitude, and (**c**) phase shift of the second eigenmode.

To demonstrate the magnetic nature of the particles, we performed bimodal magnetic force microscopy on the sample surface. This single-pass method [37] proved to be more sensitive to magnetic forces than conventional magnetic force microscopy which exploits the lift mode and can facilitate the detection of superparamagnetic nanoparticles [38,39] and their magnetic properties. Figure 5 shows the topography (a), the amplitude of the second eigenmode (b), and the second eigenmode phase (c) images. While the topography and amplitude images exhibit the local structure of the polymer, the second eigenmode phase image clearly reveals the location of the magnetic particles buried within the material (see arrows, Figure 5). The magnetic interaction of these particles with the magnetized tip (coating) of the cantilever is characterized by the low phase values in comparison with the values of their surroundings. This finding proves both the presence of the magnetic nanoparticles as part of the self-assembled microphase-separated BCP film, but also the potential local addressability of the individual magnetic particles, which will be of use for a variety of electronic and magnetic devices.

## 6. Conclusions

Within the present study, the self-assembly of FePt nanoparticles with a PMMA shell in an ultrahigh molecular weight block copolymer, PS-*b*-PMMA, was investigated. The PMMA-coated FePt nanoparticles were predominantly (96%) located within the lamellar PMMA domain within the microphase-separated film of PS-*b*-PMMA, as shown by TEM measurements. From these investigations, it could be concluded that the relatively small diameter of the nanoparticles (6.1 ± 0.6 nm) compared to the width of the PMMA domain (19 ± 3 nm) did not lead to a structural change of the block copolymer morphology. When values of 0.5 wt% were reached, a negative influence of the increasing particle concentration on the order of the lamellar morphology was observed in the TEM. The SAXS data supported the one obtained from TEM, which indicated a constant domain size of the microphase-separated lamellae with the addition of the nanoparticles. To study the magnetic addressability of the FePt nanoparticles as part of the PMMA domain within the microphase-separated PS-*b*-PMMA thin film, bimodal magnetic force microscopy proved the magnetic response of the particles within the morphology. The present study provides insights into the influence and structural changes of FePt nanoparticle concentrations on the self-assembly of ultrahigh molar mass block copolymers. The co-self-assembly of such magnetic nanoparticles and high molecular weight polymers can pave the way for electric and magnetic devices with lamellar stacks. Further studies should be performed to investigate the effect of polymer chain length and the grafting density on the particle surface. This strategy could presumably lead to a higher loading of particles without disturbing the order of the block copolymer morphology, and thus increase the magnetic response of the nanocomposite PMMA domain.

## Data Availability

Not applicable.

## Supplementary Materials

The following supporting information can be downloaded at: https://www.mdpi.com/article/10.3390/ma16165503/s1, Figure S1: overview of more TEM images of PS-*b*-PMMA with a rising amount of FePt NPs from 0.0 wt% to 1.0 wt% from top to the bottom. Inside the morphology, the PS domains appear electron opaque (dark) and the PMMA domains are light. The NPs are represented by dark dots.

## References

[1] Seifert J., Roitsch S., Schmidt A.M. (2021). Covalent Hybrid Elastomers Based on Anisotropic Magnetic Nanoparticles and Elastic Polymers. Appl. Polym. Mater..

[2] Dai Q., Berman D., Virwani K., Frommer J., Jubert P.O., Lam M., Topuria T., Imaino W., Nelson A. (2010). Self-Assembled Ferrimagnet Polymer-Composites for Magnetic Recording Media. Nano Lett..

[3] Haryono A., Binder W.H. (2006). Controlled arrangement of nanoparticle arrays in block-copolymer domains. Small.

[4] Konefal M., Cernoch P., Patsula V., Pavlova E., Dybal J., Zaleski K., Zhigunov A. (2021). Enhanced Ordering of Block Copolymer Thin Films upon Addition of Magnetic Nanoparticles. Appl. Mater. Interfaces.

[5] Kang S., Ryu D., Ringe E., Hickey R.J., Park S.J. (2020). Nanoparticle-Induced Self-Assembly of Block Copolymers into Nanoporous Films at the Air-Water Interface. ACS Nano.

[6] Xia S.L., Song L., Chen W., Korstgens V., Opel M., Schwartzkopf M., Roth S.V., Muller-Buschbaum P. (2019). Printed Thin Diblock Copolymer Films with Dense Magnetic Nanostructure. Appl. Mater. Interfaces.

[7] Mendoza C., Nirwan V.P., Fahmi A. (2023). Nanofabrication of hybrid nanomaterials: Macroscopically aligned nanoparticles pattern via directed self-assembly of block copolymers. J. Appl. Polym. Sci..

[8] Kao J., Thorkelsson K., Bai P., Rancatore B.J., Xu T. (2013). Toward functional nanocomposites: Taking the best of nanoparticles, polymers, and small molecules. Chem. Soc. Rev..

[9] Kim B.J., Chiu J.J., Yi G.R., Pine D.J., Kramer E.J. (2005). Nanoparticle-induced phase transitions in diblock-copolymer films. Adv. Mater..

[10] Lin Y., Boker A., He J.B., Sill K., Xiang H.Q., Abetz C., Li X.F., Wang J., Emrick T., Long S. (2005). Self-directed self-assembly of nanoparticle/copolymer mixtures. Nature.

[11] Misner M.J., Skaff H., Emrick T., Russell T.P. (2003). Directed deposition of nanoparticles using diblock copolymer templates. Adv. Mater..

[12] Pietsch T., Müller-Buschbaum P., Mahltig B., Fahmi A. (2015). Nanoporous Thin Films and Binary Nanoparticle Superlattices Created by Directed Self-Assembly of Block Copolymer Hybrid Materials. Appl. Mater. Interfaces.

[13] Cao W., Yin S.S., Bitsch M., Liang S.Z., Plank M., Opel M., Scheel M.A., Gallei M., Janka O., Schwartzkopf M. (2022). In Situ Study of FePt Nanoparticles-Induced Morphology Development during Printing of Magnetic Hybrid Diblock Copolymer Films. Adv. Funct. Mater..

[14] Darling S.B., Yufa N.A., Cisse A.L., Bader S.D., Sibener S.J. (2005). Self-organization of FePt nanoparticles on photochemically modified diblock copolymer templates. Adv. Mater..

[15] Aldakheel F., Ntetsikas K., Yudhanto A., Lubineau G., Hadjichristidis N. (2023). In Situ Formation of Silica Nanoparticles Decorated with Well-Defined Homopolymers and Block Copolymers. Appl. Polym. Mater..

[16] Xu X.Y., Gao Y.T., Wang Y.Y., Zhou Y.S., Xiong B.J., Zhu J.T. (2022). Surfactant Mediated Microphase Separation in Miscible Block Copolymer of Poly(4-vinyl pyridine-b-hydroxybutylacrylate). Chin. J. Polym. Sci..

[17] Song D.P., Li C., Li W.H., Watkins J.J. (2016). Block Copolymer Nanocomposites with High Refractive Index Contrast for One-Step Photonics. Acs Nano.

[18] Song D.P., Li C., Colella N.S., Xie W.T., Li S.K., Lu X.M., Gido S., Lee J.H., Watkins J.J. (2015). Large-Volume Self-Organization of Polymer/Nanoparticle Hybrids with Millimeter-Scale Grain Sizes Using Brush Block Copolymers. J. Am. Chem. Soc..

[19] Hammond M.R., Dietsch H., Pravaz O., Schurtenberger P. (2010). Mutual Alignment of Block Copolymer-Magnetic Nanoparticle Composites in a Magnetic Field. Macromolecules.

[20] Kim J., Green P.F. (2010). Directed Assembly of Nanoparticles in Block Copolymer Thin Films Role of Defects. Macromolecules.

[21] Chiu J.J., Kim B.J., Kramer E.J., Pine D.J. (2005). Control of nanoparticle location in block copolymers. J. Am. Chem. Soc..

[22] Bockstaller M.R., Lapetnikov Y., Margel S., Thomas E.L. (2003). Size-selective organization of enthalpic compatibilized nanocrystals in ternary block copolymer/particle mixtures. J. Am. Chem. Soc..

[23] Ma L., Huang H.J., Ercius P., Alexander-Katz A., Xu T. (2022). Symmetry-Breaking and Self-Sorting in Block Copolymer-Based Multicomponent Nanocomposites. ACS Nano.

[24] Matsen M.W., Thompson R.B. (2008). Particle distributions in a block copolymer nanocomposite. Macromolecules.

[25] Yeh S.W., Wei K.H., Sun Y.S., Jeng U.S., Liang K.S. (2005). CdS nanoparticles induce a morphological transformation of poly(styrene-b-4-vinylpyridine) from hexagonally packed cylinders to a lamellar structure. Macromolecules.

[26] Huh J., Ginzburg V.V., Balazs A.C. (2000). Thermodynamic behavior of particle/diblock copolymer mixtures: Simulation and theory. Macromolecules.

[27] Thompson R.B., Ginzburg V.V., Matsen M.W., Balazs A.C. (2001). Predicting the mesophases of copolymer-nanoparticle composites. Science.

[28] Hickey R.J., Haynes A.S., Kikkawa J.M., Park S.J. (2011). Controlling the Self-Assembly Structure of Magnetic Nanoparticles and Amphiphilic Block-Copolymers: From Micelles to Vesicles. J. Am. Chem. Soc..

[29] Kim B.S., Taton T.A. (2007). Multicomponent nanoparticles via self-assembly with cross-linked block copolymer surfactants. Langmuir.

[30] Zhao Y., Thorkelsson K., Mastroianni A.J., Schilling T., Luther J.M., Rancatore B.J., Matsunaga K., Jinnai H., Wu Y., Poulsen D. (2009). Small-molecule-directed nanoparticle assembly towards stimuli-responsive nanocomposites. Nat. Mater..

[31] Li Q., Wu L.H., Wu G., Su D., Lv H.F., Zhang S., Zhu W.L., Casimir A., Zhu H.Y., Mendoza Garcia A. (2015). New Approach to Fully Ordered fct-FePt Nanoparticles for Much Enhanced Electorcatalysis in Acid. Nano Lett..

[32] Fulmer G.R., Miller A.J.M., Sherden N.H., Gottlieb H.E., Nudelman A., Stoltz B.M., Bercaw J.E., Goldberg K.I. (2010). NMR Chemical Shifts of Trace Impurities: Common Laboratory Solvents, Organics, and Gases in Deuterated Solvents Relevant to the Organometallic Chemist. Organometallics.

[33] Butt H.J., Jaschke M. (1995). Calculation of Thermal Noise in Atomic-Force Microscopy. Nanotechnology.

[34] Gallei M., Schmidt B.V.K.J., Klein R., Rehahn M. (2009). Defined Poly[styrene-block-(ferrocenylmethyl methacrylate)] Diblock Copolymers via Living Anionic Polymerization. Macromol. Rapid Commun..

[35] Appold M., Gallei M. (2019). Bio-Inspired Structural Colors Based on Linear Ultrahigh Molecular Weight Block Copolymers. Appl. Polym. Mater..

[36] Hamley I.W., Castelletto V. (2004). Small-angle scattering of block copolymers in the melt, solution and crystal states. Prog. Polym. Sci..

[37] Li J.W., Cleveland J.P., Proksch R. (2009). Bimodal magnetic force microscopy: Separation of short and long range forces. Appl. Phys. Lett..

[38] Dietz C., Herruzo E.T., Lozano J.R., Garcia R. (2011). Nanomechanical coupling enables detection and imaging of 5 nm superparamagnetic particles in liquid. Nanotechnology.

[39] Stühn L., Auernhammer J., Dietz C. (2019). pH-depended protein shell dis- and reassembly of ferritin nanoparticles revealed by atomic force microscopy. Sci. Rep..

