# Supplementary material for: Self-Assembly of Polymer-Modified FePt Magnetic Nanoparticles and Block Copolymers
Source: Materials (Basel). 2023 Aug 8;16(16):5503. doi: 10.3390/ma16165503 (PMC10455748; doi:10.3390/ma16165503)
Supplement: Supplementary file 1 [file materials-16-05503-s001.zip › materials-2521783-supplementary.pdf]

Self-Assembly of Polymer-Modified FePt Magnetic Nanoparticles and Block Copolymers

Frank Hartmann [1], Martin Bitsch [1], Bart-Jan Niebuur [2], Marcus Koch [2], Tobias Kraus [2,3], Christian Dietz [4], Robert W. Stark [4], Christopher R. Everett [5], Peter Müller-Buschbaum [5,6], Oliver Janka [7] and Markus Gallei [1,8,*]

1 Polymer Chemistry, Faculty of Natural Sciences and Technology, Saarland University, Campus C4 2, 66123 Saarbrücken, Germany; frank.hartmann@uni-saarland.de (F.H.); martinbitsch@live.de (M.B.)
2 INM—Leibniz-Institute for New Materials, Campus D2 2, 66123 Saarbrücken, Germany; bart-jan.niebuur@leibniz-inm.de (B.-J.N.); marcus.koch@leibniz-inm.de (M.K.); tobias.kraus@leibniz-inm.de (T.K.)
3 Colloid and Interface Chemistry, Faculty of Natural Sciences and Technology, Saarland University, Campus D2 2, 66123 Saarbrücken, Germany
4 Physics of Surfaces, Institute of Materials Science, Technical University of Darmstadt, Peter-Grünberg-Straße 2, 64287 Darmstadt, Germany; dietz@pos.tu-darmstadt.de (C.D.); stark@pos.tu-darmstadt.de (R.W.S.)
5 Chair for Functional Materials, Department of Physics, TUM School of Natural Sciences, Technical University of Munich, James-Franck-Straße 1, 85748 Garching, Germany; christopher.everett@ph.tum.de (C.R.E.); muellerb@ph.tum.de (P.M.-B.)
6 Heinz Maier-Leibnitz Zentrum (MLZ), Technical University of Munich, Lichtenbergstraße 1, 85748 Garching, Germany
7 Inorganic Solid-State Chemistry, Faculty of Natural Sciences and Technology, Saarland University, Campus C4 1, 66123 Saarbrücken, Germany; oliver.janka@uni-saarland.de
8 Saarene, Saarland Center for Energy Materials and Sustainability, Campus C4 2, 66123 Saarbrücken, Germany
* Correspondence: markus.gallei@uni-saarland.de

Supporting Information

Nanoparticle synthesis and modification

Synthesis of FePt NPs: The synthesis of the FePt NPs was carried out according to the protocol from Q. Li et al. [31] 5 mL octadecene, 0.13 g Pt(acac)$_2$, 0.7 mL oleylamine and 0.65 mL oleic acid were added to a dry three-necked round bottom flask equipped with a KPG stirrer and an argon inlet. Afterwards the flask was heated up to 120 °C and degassed under an argon flow for at least 30 min. Then 0.11 mL of iron pentacarbonyl was added. In the next step, the temperature was increased to 220 °C with a heating rate of 5 K min^{-1} and kept for 1 h. After cooling to room temperature, 15 mL 2-propanol was added and the NPs were collected by centrifugation (8500 rpm). In addition, the particles were redispersed in hexane, mixed with 15 mL ethanol and again separated by centrifugation (8500 rpm). For further usage, the FePt NPs were redispersed in 30 mL of dry toluene.

Synthesis of 2-bromo-2-methyl-N-(3-(triethoxysilyl) propyl) propenamide: For the synthesis of 2-bromo-2-methyl-*N*-(3-(triethoxysilyl) propyl) propenamide 150 mL of dry toluene, 1.11 mL triethylamine (TEA) and 1.87 mL APTES were transferred in a dry 250 mL Schlenk flask. After cooling the reaction mixture to 0 °C with an ice bath, 0.99 mL 2-bromoisobutyrylbromide were added dropwise over 30 min. After the complete addition the reaction mixture was stirred for 2 h at 0 °C and then at room temperature overnight. To isolate the final product, the reaction mixture was filtrated and the solvent evaporated under reduced pressure. Finally, a slightly yellowish oil was obtained.

Modification of FePt NPs with 2-bromo-2-methyl-N-(3-(triethoxysilyl) propyl) propenamide: 30 mL of a FePt-NP dispersion in toluene was added to a round bottom schlenk flask equipped with a mechanical stirrer under an argon atmosphere. Subsequently, 0.1 mL TEA and 0.2 mL 2-bromo-2-methyl-*N*-(3-(triethoxysilyl) propyl) propenamide were added and the mixture was stirred for 48 h at room temperature. Afterwards, 20 mL petroleum ether were added and the NPs were separated by centrifugation (8500 rpm), redispersed in toluene and re-precipitated by petroleum ether. This step was repeated for two times to remove unreacted initiator. After drying, the particles were redispersed in 25 mL of anisole.

Surface functionalization with poly(methylmethacrylate) (PMMA): 25 mL of the modified FePt NPs dispersion were added to a three-necked round bottom Schlenk flask equipped with a mechanical stirrer and an argon inlet. After the addition of 10 mL methyl methacrylate (MMA) and 3 µL *tert*-butyl-α-bromisobutyrate, the dispersion was heated up to 90 °C. The polymerization was initiated by the addition of 2 mL of a [Cu(I)(PMDETA)Cl] solution (2 M in anisole). After 4 h the mixture was precipitated in an excess of methanol and separated by filtration. The precipitate was redispersed in THF, separated by centrifugation (23000 rpm) and washed three times with THF and two times with toluene. Finally, FePt NPs coated with PMMA were dispersed in toluene.

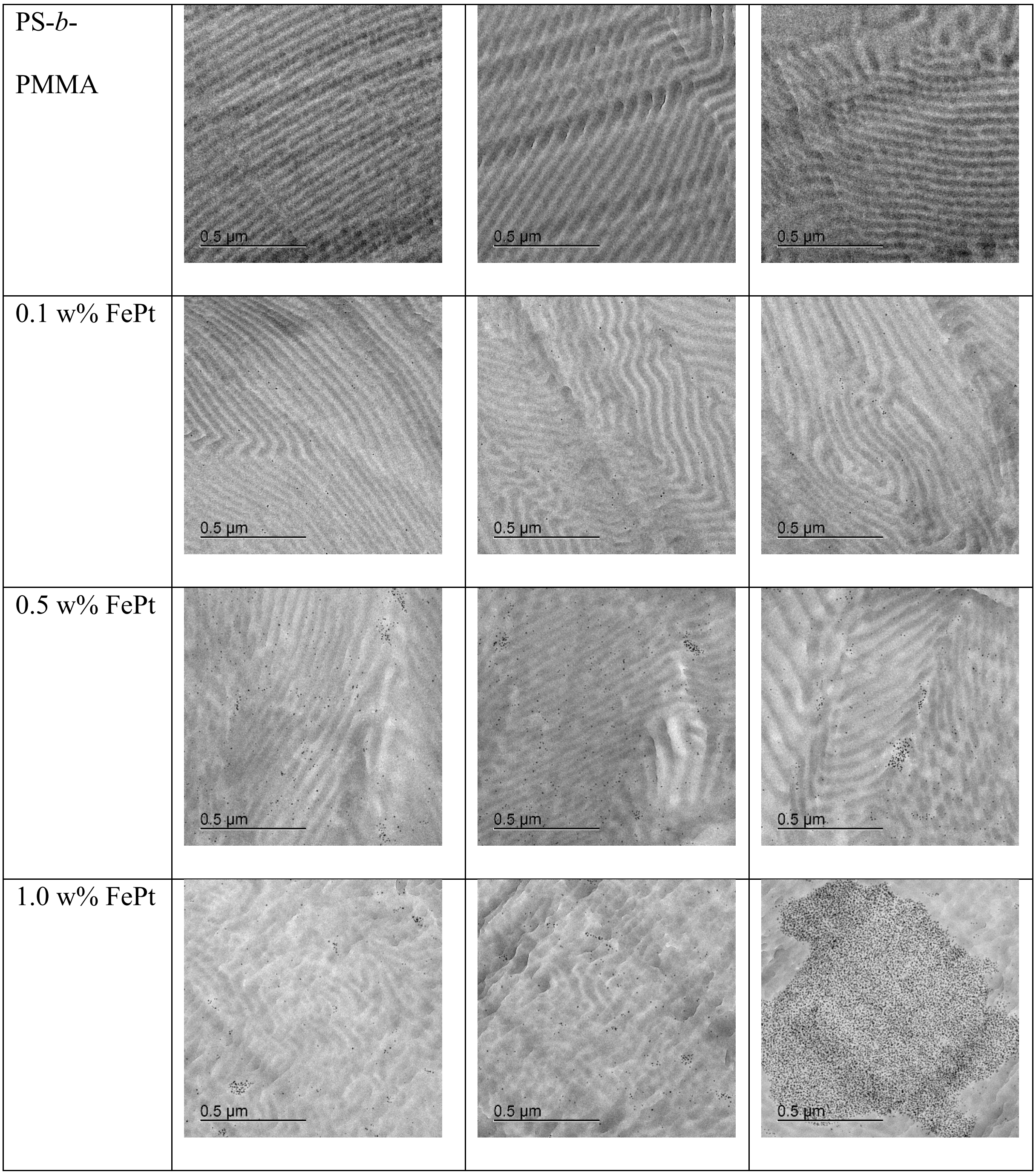

Figure S1: Overview of more TEM images of PS-*b*-PMMA with a rising amount of FePt NPs from 0.0 wt% to 1.0 w% from top to the bottom. Inside the morphology the PS domains appear electron opaque (dark) and the PMMA domains are light. The NPs are represented by the dark dots.
